# Report of Diseases from October 24th to November 24th

**Published:** 1814-12

**Authors:** John Want

**Affiliations:** 11, North Crescent


THE
### Medical and Physical Journal.

6 OF VOL. XXXII.] DECEMBER, 1814. [NO. 190.

"For many fortunate discoveries in medicine, and for the detection of nume-
"rous errors, the world is indebted to the rapid circulation of Monthly
"Journals; and there never existed any work to which the faculty in
"EUROPE and AMERICA were under deeper obligations than to the
"Medical and Physical Journal of London, now forming a long, but an
"invaluable series."—RUSH.

*For the Medical and Physical Journal.*

### REPORT *of* DISEASES *from* OCTOBER 24*th to* NOVEMBER 24*th*, BY MR. WANT.

TYPHUS FEVER has occurred in far greater numbers than I have known for several years past. Some of the cases, as may be expected, were slight, and easily subdued by the ordinary remedies; while many have been of a malignant and fatal nature. A few years since an unusual prevalence of Typhus was attributed to scarcity; but as this happily does not exist at present, we must look to other sources for its origin.

SCARLATINA continues to prevail, and has much increased in severity since the last report. A friend has communicated to me four fatal cases, which came under his care in succession. On enquiry respecting the treatment pursued, I learned that Peruvian bark had been given in large doses, the pernicious effects of which I have too often had occasion to witness. It is very questionable whether any known *medicine* be deserving of confidence in these cases. But cold ablution of the skin rarely fails to mitigate the symptoms, and as rarely fails to cure. From experience I should be inclined to say, that cold water drank and applied externally, while the heat of the skin is above the natural standard, are the only remedies on which dependance can be placed. The dropsical swelling of the legs, often consequent to this disease, may generally be removed by immersing the parts in water, as hot as can be borne, every night at bedtime.

In a poor girl about eight years of age, notwithstanding the febrile symptoms were each time removed by the ablution of the skin, they continued to recur for the space of three three weeks. Mention was made of sore throat, but, as no great stress was laid on it, a common gargle was enjoined, which was used without benefit. On examining the fauces, the tonsils were found to be very much enlarged, and externally a swelling existed behind each angle of the jaw, corresponding in situation with the tumid glands. I recommended an incision to be made by a scalpel through their substance, which, in general, I have experienced to give instant relief. The incision was opposed, and a blister was applied to each angle of the jaw. This produced an abatement of the difficulty of swallowing; but no permanent good was effected until a quantity of matter was evacuated by the rupture of an abscess in each tonsil. Both the fever and the difficulty of swallowing immediately disappeared.

Among the inflammatory complaints, Catarrh, Pneumonia, and Measles, hold a distinguished place; but nothing deserving of remark has been observed.

Rheumatic cases have fallen under my notice in great abundance. The disease is now very prevalent. In its acute form, I have seen much benefit arise from the use of Colchicum. In two severe cases of this affection, after it had degenerated into that species which many would call Rheumatic Gout,* the colchicum removed, in one night, an ailment of upwards of two months' continuance. In Chronic Rheumatism it is not so effectual, but is still serviceable in many cases.

A variety of remedies were ineffectually tried with a view to relieve the intense pain of a severe Scald in both legs, occasioned by boiling water. Lime-water and linseed oil, cold water, saturnine and opiate lotions, terebinthinate applications, and poultices, were employed in succession, with large doses of opium internally. Observing that some parts of the limb were covered with innumerable *small* vesicles, and that the pain was more intense in them than in other places, I carefully opened the whole of them by the scissars, or by puncturing with a needle where they were too small to be cut. The effect of this practice was almost immediate in removing the agony of the pain. The limbs were then enveloped in a linseed-meal poultice, and the patient experienced comparatively little pain from that time.

Paralysis of the Wrists, consequent to working in lead, was observed in a man *who makes bougies, and spreads adhe-*

* An inflammatory and painful swelling in the larger joints, flying from one to the other, but *without fever.*

*sive plaster for the shops.* The patient has, during the last seven years, been attacked as many times with Colica Pictonum. The use of the wrists was lost on the first invasion of the complaint.

Being incapable of taking opium from its effects on the head, the only remedy which gives him relief from pain is large potations of gin, which he never takes at any other time. He ascribes his sufferings solely to the fumes of the litharge plaster, and to the composition used in making bougies, both of which he prepares in large quantities.

The term Pseudo-syphilis has been used to express the character of a train of symptoms observed in a gentleman practising in the Inner Temple, which so closely resembled syphilis in their progress, that much caution was necessary in forming a correct judgment of their nature. It is much to be feared that many are made victims to the confounding of these two diseases. Mercury, the remedy of the one, generally aggravates the other; and, what is of infinitely greater moment, it often happens that the increase of the symptoms, occasioned solely by it, is supposed to be the mark of virulence in the disease, requiring a more vigorous prosecution of the mercurial course. I have recently witnessed two cases, where, in the practice of others, exfoliations of the lower jaw bones have been produced by the imprudent use of mercury in diseases mistaken for venereal.

In this gentleman, ulcerations were seen on the penis, but on minute examination, they were discovered to be without the hardened base common to venereal chancres; and they healed by the application of lunar caustic in solution in about a week. After these, another appeared on the prepuce, which was treated in the same manner, and without my advice. A solitary gland in the left groin was enlarged, and continues so at this time, to the great annoyance of my patient, who is still not without apprehensions that it may prove to be venereal.

Within these ten days, a period of six weeks having elapsed since the first attack, an ulcerated sore throat, of a suspicious nature, appeared, unaccompanied by febrile symptoms. The pulse was accelerated. He was much troubled with head-ach, and occasional bleeding from the nose, and the prostration of strength was so great as to prevent his leaving the house. A mixture of sulphuric acid was directed three or four times a-day, with a common gargle. The throat gradually amended under this treatment, but a new symptom arose in its stead. A plentiful eruption of copper-coloured spots, of various sizes, were seen in most parts of the body; an erysipelatous inflammation attacked the pre-

 puce puce and glans; and the seat of the former ulcerations was much indurated. From attention to the history of the case, I still thought myself justified in retaining my original opinion, and therefore made no alteration in the treatment. The spots were dying away; and the patient went to Margate to make trial of sea-bathing. The plummer's pill was the only remedy prescribed for him. He returned in the course of a month, with evident amendment of health. The eruption is still visible, but what the results of the case may be is yet uncertain.

I was desired yesterday evening to be present at the dissection of a child *supposed* to have died of HYDROCEPHALUS. He had been drooping for the space of six months, but no remedies, excepting occasional purgatives, had been resorted to. About a fortnight before his death, he was seized with convulsions: on recovering from them, he complained of pain in his head, and soon relapsed into a state of delirium and coma. From this he was occasionally roused by the violence of the pain, which manifested itself by loud screamings. The brain and its membranes were found to be greatly inflamed, and the substance of the former was unusually firm. A very small quantity of fluid comparatively was found effused into the ventricles. A modern author* affirms that Hydrocephalus is *never* the consequence of inflammation of the brain or its meninges, which is too obviously fallacious to require refutation; but the preceding case is a proof that inflammation of the brain may be *mistaken* for that disease, which should operate as a caution to practitioners in deciding on the treatment to be pursued.

11, *North Crescent.* JOHN WANT,

---

*For the Medical and Physical Journal.*

